# Embedding Patient Priorities Care in an Age-Friendly Health System

**DOI:** 10.1093/geroni/igaa057.2401

**Published:** 2020-12-16

**Authors:** Aanand Naik, Lea Kiefer, Angela Catic, Lillian Dindo

**Affiliations:** 1 Center for Innovations in Quality, Effectiveness, and Safety (iQuest), Houston, Texas, United States; 2 Michael E. DeBakey VA Medical Center, Houston, Texas, United States; 3 Baylor College of Medicine, Houston, Texas, United States

## Abstract

Background: Patient Priorities Care (PPC) is an innovative approach to improving care for older adults with multiple morbidities. We developed a PPC training program for healthcare professionals and describe preliminary results. Methods: We implemented PPC in a geriatrics clinic. 20 staff and trainees participated on 1) how to identify patient priorities, 2) documentation in the electronic health record (EHR), and 3) strategies to align care with priorities; and received case-based audit and feedback. Results: 250 patients participated in PPC encounters. The EHR template was subsequently integrated within an Age Friendly Health System (AFHS) note. Clinicians have integrated this AFHS template for all encounters. Conclusion: PPC is a feasible approach to the care of older adults with multiple morbidities following a structured clinician training program. PPC can be effectively incorporated into the “Matter Most” component of AFHS.

